# Effects of Unilateral Cochlear Implantation on Balance Control and Sensory Organization in Adult Patients with Profound Hearing Loss

**DOI:** 10.1155/2015/621845

**Published:** 2015-10-25

**Authors:** Cécile Parietti-Winkler, Alexis Lion, Bettina Montaut-Verient, Rémy Grosjean, Gérome C. Gauchard

**Affiliations:** ^1^Department of Otorhinolaryngology, Head and Neck Surgery, University Hospital, 29 avenue du Maréchal de Lattre de Tassigny, 54035 Nancy Cedex, France; ^2^Faculty of Medicine, Université de Lorraine, 9 avenue de la Forêt de Haye, CS 50184, 54505 Vandoeuvre-lès-Nancy, France; ^3^EA 3450 DevAH, Development, Adaptation and Disadvantage, Faculty of Medicine, Université de Lorraine, CS 50184, 54505 Vandoeuvre-lès-Nancy, France; ^4^Sports Medicine Research Laboratory, Luxembourg Institute of Health, 1460 Luxembourg, Luxembourg; ^5^UFR STAPS, Faculty of Sport Sciences, Université de Lorraine, 30 rue du Jardin Botanique, CS 30156, 54603 Villers-lès-Nancy, France

## Abstract

Many studies were interested in the consequence of vestibular dysfunction related to cochlear implantation on balance control. This pilot study aimed to assess the effects of unilateral cochlear implantation on the modalities of balance control and sensorimotor strategies. Posturographic and vestibular evaluations were performed in 10 patients (55 ± 20 years) with profound hearing loss who were candidates to undergo unilateral multichannel cochlear implantation. The evaluation was carried out shortly before and one year after surgery. Posturographic tests were also performed in 10 age-matched healthy participants (63 ± 16 years). Vestibular compensation was observed within one year. In addition, postural performances of the patients increased within one year after cochlear implantation, especially in the more complex situations, in which sensory information is either unavailable or conflicting. Before surgery, postural performances were higher in the control group compared to the patients' group. One year after cochlear implantation, postural control was close to normalize. The improvement of postural performance could be explained by a mechanism of vestibular compensation. In addition, the recovery of auditory information which is the consequence of cochlear implantation could lead to an extended exploration of the environment possibly favoring the development of new balance strategies.

## 1. Introduction

Cochlear implantation aims to restore hearing ability and to improve the quality of life (QoL) of patients with severe deafness [1]. The surgical procedure consists in inserting a multielectrode array into the cochlea. Vestibular damage following the surgery is possible due to the anatomical proximity between the vestibular system and the cochlea. Therefore, this insertion may alter the inner ear and may induce vestibular disorders. Indeed, up to 75% of patients undergoing cochlear implant surgery report postoperative vestibular symptoms such as vertigo, dizziness, or imbalance [2–8].

Maintaining equilibrium in upright stance requires the central processing of input signals from the visual, somatokinesthetic, and vestibular systems, leading to a context-specific motor response through the adjustments of static and dynamic postures [9, 10]. Therefore, the alteration of the inner ear by the cochlear implantation may generate postural disorders just after the surgery and after the activation of the cochlear implant [11]. Despite possible inner ear damage induced by the surgery, improvement of the postural stability has been observed even two years after cochlear implantation [2, 12]. Postural improvement after implantation may be induced by a vestibular compensation of a previously uncompensated vestibular lesion [2]. However, several studies showed that postural control of teenagers and adults remains impaired after implantation compared to healthy control subjects, without any effect of hearing restoration even 5 years after implantation [13–16]. The effect on the postural control of the activation of the cochlear implant (CI) has been evaluated in adults between 6 and 8 weeks after surgery and shows that postural control is improved in the more demanding postural situations when the CI is switched on [17].

The characterization of the possible vestibular effects induced by cochlear implantation is well performed by the classical tests (e.g., caloric test, rotary test, and video head impulse test) [6, 7]. The current absence of consensus concerning the outcome of cochlear implantation on postural control does not allow drawing any conclusions for their potential effects. This pilot study aimed to assess the effects of cochlear implantation on the modalities of balance control and sensorimotor strategies.

## 2. Material and Methods

### 2.1. Participants

This prospective study (one-year follow-up study) was conducted at the Nancy University Hospital (France) and involved 10 patients (CI group, median age = 55 ± 20 years, age range = 27 to 72 years) with profound hearing loss scheduled to receive unilateral multichannel cochlear implant (Oticon Medical-Neurelec, Vallauris, France/Cochlear Macquarie University, New South Wales, Australia) and 10 healthy participants (control group, age median = 63 ± 16 years, age range = 24 to 71 years). The indication for unilateral cochlear implantation was bilateral profound sensorineural hearing loss with no benefit from hearing aids. All participants were free from any central nervous system disease and presented no orthopedic disorders either of the trunk or the lower limbs that could affect postural performance. All participants gave written consent prior to this pilot study. This pilot study was conducted to examine the feasibility of this approach used in a larger scale study which was approved by the local ethics committee (*Comité de Protection des Personnes de Lorraine*).

### 2.2. Data Collection

All patients were submitted to hearing, gaze control, and posturographic evaluations two days before and one year after unilateral cochlear implantation. Healthy participants, who were free from ENT disorders, were only submitted to posturographic testing.

#### 2.2.1. Hearing Performance Evaluation

Lafon's cochlear lists, which were used to evaluate hearing ability [1], are composed of 17 triphonemic French words; the percentage of phonemes recognized among the 51 phonemes present in the lists gives an intelligibility score (hearing performance) for each subject. Scores without lip-reading were obtained in quiet at 70 dB SPL. One year after surgery, CI users were tested with their own processor programmed with their normal everyday processing strategy and electrode configuration.

#### 2.2.2. Visuooculomotor and Vestibular Evaluation

The visuooculomotor and vestibular assessments were performed with videonystagmography (Synapsys, Marseille, France) (see details in [18, 19]). The visuooculomotor tests included the evaluation of smooth pursuit and saccades. Spontaneous nystagmus without visual fixation (in darkness) was also recorded. The caloric test, which remains the gold standard to evaluate the degree of vestibular asymmetry, was performed according to the bithermal caloric test protocol, with water infusion of the ear canal for 30 seconds. The subject was in the supine position with the head flexed 30°. Vestibular areflexia was characterized by an absence of caloric response on the side of the affected labyrinth, while vestibular hyporeflexia was determined by a decreased response of more than 20% on the side of the affected labyrinth [20]. For the rotary test, the patients sat in a rotary chair (MED4, Synapsys) with opened eyes in a dark room, and the plane of the lateral semicircular canal was positioned perpendicular to the axis of rotation. The rotary chair test protocol was a pendular test, consisting in seven sinusoidal oscillations around an earth-vertical axis at 0.22 Hz frequency, with increasing then decreasing amplitude. The highest instantaneous velocity of the stimulus was about 30°/s and the highest oscillation amplitude about 30°. Fourier analyses were performed to calculate both the slow phase eye velocity and the chair velocity. The gain measurements of the vestibuloocular reflex were determined by the ratio of the amplitudes of the eye velocity to that of the chair velocity. The directional preponderance measurements were determined by the mean slow phase eye velocity over the duration of the stimulus.

#### 2.2.3. Postural Control Evaluation

The sensory organization test (SOT, EquiTest, Clackamas, OR) aims to evaluate a subject's ability to maintain balance control in six different conditions which are repeated three times during 20 seconds. During these trials, the displacements of the center of foot pressure are recorded. Postural control is challenged by using a technique commonly referred to as sway-referenced, which involves tilting the support surface and/or the visual surround to directly follow the anterior-posterior sways of the subject's center of gravity [21]. Vision is not challenged in conditions 1 (C1) and 4 (C4). Eyes are closed in conditions 2 (C2) and 5 (C5). The visual surround may move in conditions 3 (C3) and 6 (C6). The support surface may move in conditions 4 to 6. These six conditions increase in difficulty and were not randomized. The theoretical limit of stability is based on the individual's height and size of the base of support. The following formula was used to calculate the equilibrium score: [12.5° − ((*θ*_max_ – *θ*_min_)/12.5°)] × 100, where *θ*_max_ indicates the greatest anteroposterior center of gravity sway angle displayed by the subject while *θ*_min_ indicates the lowest anteroposterior center of gravity sway angle. Lower sways lead to a higher score, indicating a better balance control performance (a score of 100 represents no sway, while 0 indicates sway that exceeds the limit of stability, resulting in a fall). Equilibrium scores (ES) were calculated for every condition (C1^ES^ to C6^ES^). A composite equilibrium score (C^ES^) was calculated by adding the average scores from conditions 1 and 2 and the ES from each trial of conditions 3 to 6, and finally dividing that sum by the total number of trials [21–23].

The participants were requested to stand upright and barefoot at mark level on the support surface to control stance width, remaining as still as possible, breathing normally, and being with their arms at their sides. They were instructed to look straight ahead at a picture located on the visual surround. To protect against falls, an operator stood within reaching distance of the participant and all wore a safety harness connected to the ceiling by two suspension straps in all test conditions.

### 2.3. Statistical Analysis

Qualitative data were expressed as percentages (%) and compared using Fisher's exact test. Quantitative data were expressed as median with interquartile range and compared using nonparametric statistical tests. The Wilcoxon test was performed to compare the results observed before and one year after cochlear implantation. A Mann-Whitney test was performed to compare the results between the CI and control groups. Statistically significant differences were accepted for a probability level of *P* < 0.05.

## 3. Results

### 3.1. Participants

Gender, etiology of deafness, age at implantation, deafness duration, side of implantation, hearing performances before and after cochlear implantation, and vestibular status before and after cochlear implantation are presented in Table 1. The implantation was performed on the right side in 7 patients and on the left side in 3 patients by the same surgeon. The CI was inserted unilaterally via the round window surgical technique for one patient and via a cochleostomy procedure for the nine remaining patients. Data on age, gender, height, weight, and body mass index for patients and controls are presented in Table 2 and no significant difference was observed between the two groups for all these parameters.

### 3.2. Visuooculomotor and Vestibular Evaluation

**Figure 1:**
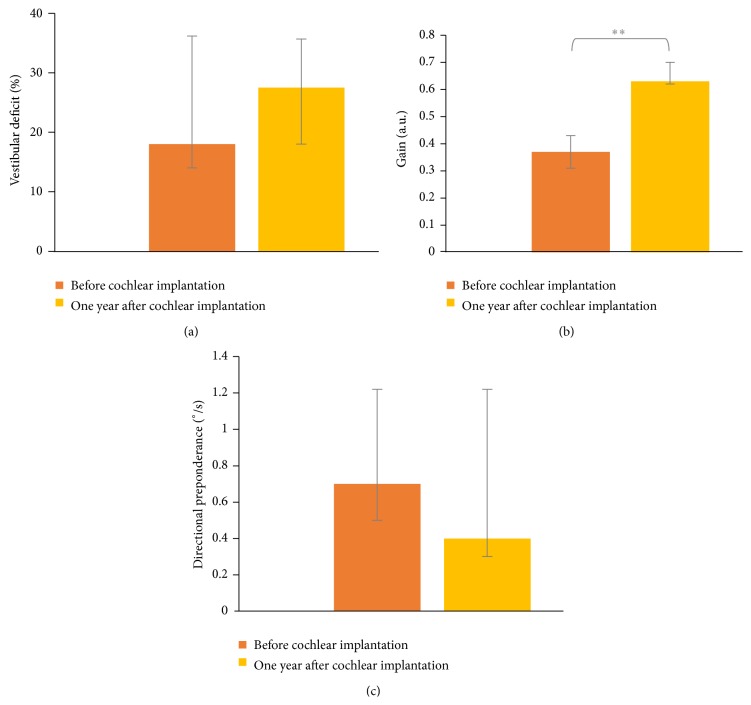
Median values and interquartile ranges of the vestibular status from the caloric test (a) and of the gain (b) and directional preponderance (c) from the rotatory chair test observed in CI group before (orange bars) and one year after cochlear implantation (yellow bars). ^*∗∗*^ *P* < 0.01.

No participant declared vertigo or dizziness. The visuooculomotor tests showed that smooth pursuit and saccades were normal for all the patients. For the gaze test, only one patient presented a spontaneous nystagmus before cochlear implantation. One year after surgery, no spontaneous nystagmus was observed. Before surgery, the caloric test showed that 1 patient had vestibular areflexia and 4 patients had vestibular hyporeflexia (Table 1). The visual fixation inhibited the nystagmus induced by the caloric test in all these patients. The surgery did not modify the vestibular status of the patients (Figure 1(a)). For the rotatory chair test, the gain (Figure 1(b)) was higher one year after cochlear implantation than before surgery (*P* = 0.005), whereas no difference was observed for the directional preponderance (Figure 1(c)).

### 3.3. Evolution of Postural Control within One Year in CI Patients

**Figure 2:**
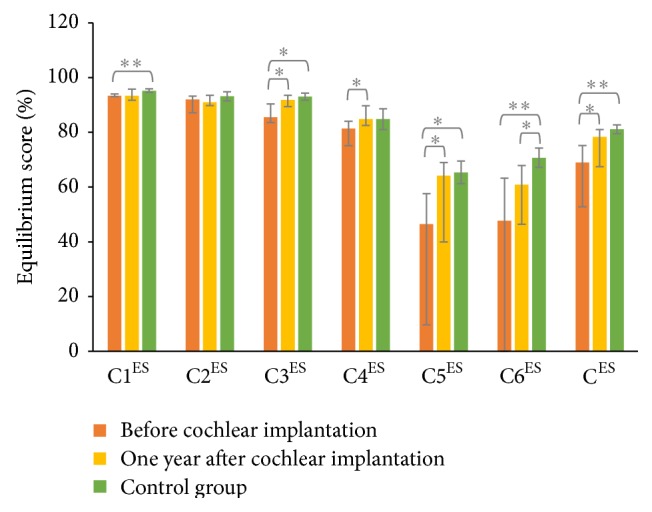
Sensory organization test: median values and interquartile ranges of the equilibrium scores (ES, in %) for the six conditions (C1^ES^ to C6^ES^) and the composite equilibrium score (C^ES^) observed in CI group before (orange bars) and one year after cochlear implantation (yellow bars) and in control group (green bars). ^*∗*^ *P* < 0.05, ^*∗∗*^ *P* < 0.01.

The evolution of postural control within one year in CI patients is showed in Figure 2. Postural control improved one year after surgery compared to before surgery. Indeed, C^ES^ was higher one year after surgery (*P* = 0.021), mainly because of higher performances in C3 (*P* = 0.025), C4 (*P* = 0.033), and C5 (*P* = 0.043). In addition, postural improvement was also observed for the patient who had vestibular areflexia (C^ES^ varying from 27 to 65% within one year).

### 3.4. Comparison of Postural Control between CI and Control Groups

Before surgery (Figure 2), CI patients had altered postural performances. Indeed, C^ES^ was lower in the CI group before surgery than in the control group (*P* = 0.010), which mainly resulted from lower performances in C1 (*P* = 0.008), C3 (*P* = 0.013), C5 (*P* = 0.015), and C6 (*P* = 0.008). One year after surgery, only one difference was observed between the CI group and the control group. Indeed, postural performances normalized in all postural conditions, except for C6 which remained significantly different between the two groups (*P* = 0.019).

## 4. Discussion

This prospective study showed that patients with unilateral cochlear implants displayed an improvement of postural performance one year after implantation compared to before surgery (even for the patient who had unilateral vestibular areflexia). The gain at the rotatory chair test, which was low before surgery, increased considerably one year after cochlear implantation. In addition, postural performances, which were altered before surgery especially in the more complex conditions, increased one year after cochlear implantation and reached the performances observed in the control group. However, a difference is still observed one year after cochlear implantation between the CI and control groups in the most challenging condition characterized by the possible simultaneous movements of the visual surrounding and the support surface.

Deaf patients, who are candidates to cochlear implantation, have normal or decreased vestibular function, vestibular dysfunction in some deaf patients being related to a joint pathology of the posterior and anterior labyrinths [3, 24, 25]. Indeed, CI candidates have often preoperative vertigo symptoms [3]. In our study, CI patients had low preoperative gain at the rotatory chair test, suggesting that vestibular function was altered before surgery and highlighting a low efficiency of vestibular compensation [26]. One hypothesis may be that this low degree of vestibular compensation is one explanatory factor of low preoperative postural performance, which is in accordance with previous observations by Magnusson et al. [25]. According to the theoretical framework of perception-action, the brain receives information from the various sensory afferents to produce movement, and the action determines the perception [27]. Applied to vestibular pathology, two things are required in order to compensate. First, the brain must receive signals from the balance organs. Thus, movements must not be avoided, because movements create the signals which are required by the brain to compensate for the injury. Second, the balance areas of the brain must be capable of change. In CI candidates, the brain is usually not damaged and is capable of plasticity. Therefore, the failure of preoperative compensation is probably induced by the isolation and the restriction of activity related to the deafness [28]. One year after cochlear implantation, patients showed no degradation of their postural performances in spite of the introduction of the electrode array in the cochlea which may increase the risk of the vestibular asymmetry to the side of the implantation. The higher values of the gain and the lower values of directional preponderance, observed in the rotatory chair test one year after cochlear implantation, demonstrate the efficiency of the vestibular compensation. This vestibular compensation is associated with a postural compensation highlighted by the absence of degradation of postural performance one year after cochlear implantation. As it had been suggested in other kinds of vestibular dysfunction, for example, in the case of vestibular schwannoma, the time-course of implementation of central adaptive mechanisms, characterized by substitution by other sensory afferences and new behavioral strategies, could lead to a recovery of balance control with an improvement in balance performance [29, 30], which are close to normal and are difficult to decompensate [31].

One year after cochlear implantation, the postural performances did not decrease. Conversely, the performances improved significantly and tended to reach normal performances. The vestibular compensation could not explain alone the major improvement of the postural performances. The restoration of the auditory information could contribute to balance regulation in CI patients according to two complementary hypotheses. On the one hand, the recovery of hearing may lead to a reorientation of CI patients in their surroundings. Indeed, equilibrium function relies on the control of posture but also on spatial orientation. The spatial orientation of the body allows a subject to perceive his/her environment and to act particularly in case of movement or destabilization [32]. Posture can thus be considered as a primary support for action [9]. Although auditory information is not considered as a fundamental signal involved in balance control, the auditory system is a perceptual system which, with vision and touch, is involved in the perception of the dynamic environment and in complex representations of 3D space. In the same way as sensory inputs are redundant and complementary to fine-tune postural control during sensory conflict situations [18], the ability to use multiple senses to perceive environmental characteristics allows a more relevant detection of objects and events, leading to an accurate orientation behaviour [33]. Indeed, recent studies showed that multisensory cues were more effective at capturing spatial attention than unimodal cues under conditions of concurrent perceptual tasks [34, 35]. The recovery of auditory function in environmental perception and orientation references after cochlear implantation could initiate motor learning and, in this way, allow the implementation of new neural networks, leading to new sensorimotor and behavioral strategies. These new sensorimotor and behavioral strategies could explain the improvement of postural control efficiency observed here, especially in the more complex postural situations. CI patients displayed new balance abilities. This could be related to the recovery of auditory information, which may participate in spatial orientation through interactions with the visual signals [36, 37]. On the other hand, cochlear implantation is known to induce an improvement in the quality of life (QoL). A multiple stepwise regression analysis showed that, after implantation, improvements in communication abilities, reduced psychological problems, and improvements of daily life abilities were key determinants to QoL improvements for CI patients [1, 38]. The QoL increase reaches a plateau at about 1.5 to 3 years after implantation [39]. The recovery of hearing may therefore reduce isolation, depressive state, restriction of activity, and the breakdown of familial or other social occupational activities. These factors could influence vestibular and postural compensations. Therefore, CI patients may interact more with the environment which may lead to enhanced sensory-motor stimulations.

The minor remaining perturbations of postural control observed one year after surgery in CI patients indicate that postural compensation is close to be completed and could justify a longer follow-up. Indeed, balance performances still improved one year after unilateral vestibular deafferentation and this improvement could be related to a reinforcement of the newly elaborated sensorimotor and behavioral strategies due to daily life activities [19]. Thus, postural performances might need more than one year to reach age-matched control level in CI patients, who have to perform simultaneously several learning processes, such as auditory rehabilitation with speech recognition learning, vestibular compensation after vestibular function degradation related to the introduction of the electrode array in the cochlea, and finally new orienting behaviour learning process after recovery of auditory perception of dynamic environment.

The main limitation of this study is the possible postural learning effect that could be observed between the two measurements in the CI group. The learning effect has been observed to last at least three months [40, 41]. However, this hypothesis is not convincing according to the sensorimotor modifications induced by surgical approach and the delay (one year) between the two posturographic evaluations. Therefore, the learning effect is probably limited. In addition, the test-retest reliability of the composite and equilibrium scores has been shown to be fairly good [42]. Another limitation of this study is the sound from the engines and from the movements of the screen or the platform during the sway-referenced conditions. It cannot be excluded that the CI patients have used these sounds to limit their movements. In addition, the EquiTest used the most extreme data samples to evaluate the postural control; this is therefore flawed as it is not fully representative of the average stability. However, this device is fairly convenient to use in a clinical setting. Finally, the study did not investigate the orientation ability of the CI patients. The results of this study should be interpreted in light of these limitations and further prospective studies are needed especially with the standardization of the CI patients according to the origin of the hearing loss and the recovery of hearing after cochlear implantation.

## 5. Conclusions

Unilateral cochlear implantation is not harmful for postural performances. Conversely, postural stability improves within one year after the cochlear implantation. This improvement could be an indirect consequence of the recovery of auditory information. Indeed, patients may be less dependent and move more, strengthening postural control. The increase of postural control performances could also take an important part in the improvement of the quality of life observed in CI patients. Vestibular and postural evaluations are important in the follow-up of the cochlear implantation. The vestibular and postural improvements should be taken into account in the decision-making process of the cochlear implantation.

## Figures and Tables

**Table 1:** Characteristics of the ten CI patients.

Patient number	Gender	Etiology of deafness	Age when implanted (in years)	Deafness duration (in years)	CI side	Caloric test		Hearing performance (% of phonemes recognized)	
						Presurgery	Postsurgery	Presurgery	Postsurgery
1	Female	Unknown	58	55	R	N	N	0	90
2	Female	Unknown	39	35	R	RH	RH	0	100
3	Female	Unknown	42	0.5	L	LH	LH	0	50
4	Female	Unknown	52	22	L	N	N	0	60
5	Female	Unknown	69	2	R	N	N	0	80
6	Female	Genetic	59	2	R	RA	RA	0	100
7	Female	Cogan's syndrome	27	2	L	N	N	10	100
8	Male	Unknown	52	18	R	N	N	10	60
9	Male	Otosclerosis	72	39	R	RH	RH	0	90
10	Male	Otosclerosis	66	8	L	RH	RH	0	100

CI side: L, left; R, right. Caloric test: LH, left hyporeflexia; N, normoreflexia; RA, right areflexia; RH, right hyporeflexia.

**Table 2:** Age and anthropometric characteristics, expressed in median associated with interquartile range (IQR), observed in cochlear implant patients (CI group) and in healthy participants (control group).

	CI group (*n* = 10)	Control group (*n* = 10)	*P* values^*∗*^
Women, *n* (%)	7 (70%)	7 (70%)	NS
Age (years), median (IQR)	55.0 (20.0)	63.0 (16.0)	NS
Height (m), median (IQR)	1.67 (0.05)	1.65 (0.05)	NS
Weight (kg), median (IQR)	64.0 (30.0)	57.5 (9.0)	NS
Body mass index (kg/m^2^), median (IQR)	23.5 (7.2)	21.1 (3.0)	NS

^*∗*^ *P* values for Fisher exact test or Mann-Whitney tests. NS: non-significant.

## References

[1] Rumeau C., Frère J., Montaut-Verient B., Lion A., Gauchard G., Parietti-Winkler C. (2014). Quality of life and audiologic performance through the ability to phone of cochlear implant users. *European Archives of Oto-Rhino-Laryngology*.

[2] Buchman C. A., Joy J., Hodges A., Telischi F. F., Balkany T. J. (2004). Vestibular effects of cochlear implantation. *Laryngoscope*.

[3] Krause E., Louza J. P. R., Hempel J.-M., Wechtenbruch J., Rader T., Gürkov R. (2008). Prevalence and characteristics of preoperative balance disorders in cochlear implant candidates. *Annals of Otology, Rhinology and Laryngology*.

[4] Krause E., Louza J. P. R., Hempel J.-M., Wechtenbruch J., Rader T., Gürkov R. (2009). Effect of cochlear implantation on horizontal semicircular canal function. *European Archives of Oto-Rhino-Laryngology*.

[5] Krause E., Louza J. P. R., Wechtenbruch J., Gürkov R. (2010). Influence of cochlear implantation on peripheral vestibular receptor function. *Otolaryngology—Head and Neck Surgery*.

[6] Vibert D., Häusler R., Kompis M., Vischer M. (2001). Vestibular function in patients with cochlear implantation. *Acta Oto-Laryngologica, Supplementum*.

[7] Batuecas-Caletrio A., Klumpp M., Santacruz-Ruiz S., Gonzalez F. B., Sánchez E. G., Arriaga M. (2015). Vestibular function in cochlear implantation: correlating objectiveness and subjectiveness. *The Laryngoscope*.

[8] Melvin T.-A. N., Santina C. C. D., Carey J. P., Migliaccio A. A. (2009). The effects of cochlear implantation on vestibular function. *Otology and Neurotology*.

[9] Massion J., Woollacott M. H., Bronstein A. M., Brandt T., Woollacott M. H. (1996). Posture and equilibrium. *Balance, Posture and Gait*.

[10] Maurer C., Mergner T., Peterka R. J. (2006). Multisensory control of human upright stance. *Experimental Brain Research*.

[11] Filipo R., Patrizi M., La Gamma R., D'Elia C., La Rosa G., Barbara M. (2006). Vestibular impairment and cochlear implantation. *Acta Oto-Laryngologica*.

[12] Abramides P. A., Bittar R. S., Tsuji R. K., Bento R. F. (2015). Caloric test as a predictor tool of postural control in CI users. *Acta Oto-Laryngologica*.

[13] Kluenter H.-D., Lang-Roth R., Guntinas-Lichius O. (2009). Static and dynamic postural control before and after cochlear implantation in adult patients. *European Archives of Oto-Rhino-Laryngology*.

[14] Bernard-Demanze L., Léonard J., Dumitrescu M., Meller R., Magnan J., Lacour M. (2014). Static and dynamic posture control in postlingual cochlear implanted patients: effects of dual-tasking, visual and auditory inputs suppression. *Frontiers in Integrative Neuroscience*.

[15] Suarez H., Angeli S., Suarez A., Rosales B., Carrera X., Alonso R. (2007). Balance sensory organization in children with profound hearing loss and cochlear implants. *International Journal of Pediatric Otorhinolaryngology*.

[16] Huang M.-W., Hsu C.-J., Kuan C.-C., Chang W.-H. (2011). Static balance function in children with cochlear implants. *International Journal of Pediatric Otorhinolaryngology*.

[17] Schwab B., Durisin M., Kontorinis G. (2010). Investigation of balance function using dynamic posturography under electrical-acoustic stimulation in cochlear implant recipients. *International Journal of Otolaryngology*.

[18] Parietti-Winkler C., Gauchard G. C., Simon C., Perrin P. P. (2008). Visual sensorial preference delays balance control compensation after vestibular schwannoma surgery. *Journal of Neurology, Neurosurgery and Psychiatry*.

[19] Parietti-Winkler C., Gauchard G. C., Simon C., Perrin P. P. (2010). Long-term effects of vestibular compensation on balance control and sensory organisation after unilateral deafferentation due to vestibular schwannoma surgery. *Journal of Neurology, Neurosurgery and Psychiatry*.

[20] Bergenius J., Magnusson M. (1988). The relationship between caloric response, oculomotor dysfunction and size of cerebello-pontine angle tumours. *Acta Oto-Laryngologica*.

[21] Nashner L. M., Peters J. F. (1990). Dynamic posturography in the diagnosis and management of dizziness and balance disorders. *Neurologic Clinics*.

[22] Black F. O., Paloski W. H., Doxey-Gasway D. D., Reschke M. F. (1995). Vestibular plasticity following orbital spaceflight: recovery from postflight postural instability. *Acta Oto-Laryngologica*.

[23] Gauchard G. C., Vançon G., Meyer P., Mainard D., Perrin P. P. (2010). On the role of knee joint in balance control and postural strategies: effects of total knee replacement in elderly subjects with knee osteoarthritis. *Gait and Posture*.

[24] Black F. O. (1977). Present vestibular status of subjects implanted with auditory prostheses. *The Annals of Otology, Rhinology & Laryngology. Supplement*.

[25] Magnusson M., Petersen H., Harris S., Johansson R. (1995). Postural control and vestibular function in patients selected for cochlear implantation. *Acta Oto-Laryngologica. Supplementum*.

[26] Parietti-Winkler C., Gauchard G. C., Simon C., Perrin P. P. (2011). Pre-operative vestibular pattern and balance compensation after vestibular schwannoma surgery. *Neuroscience*.

[27] Berthoz A. (1997). *Le Sens du Mouvement*.

[28] Hinderink J. B., Krabbe P. F. M., van den Broek P. (2000). Development and application of a health-related quality-of-life instrument for adults with cochlear implants: the Nijmegen cochlear implant questionnaire. *Otolaryngology—Head and Neck Surgery*.

[29] Parietti-Winkler C., Gauchard G. C., Simon C., Perrin P. P. (2006). Sensorimotor postural rearrangement after unilateral vestibular deafferentation in patients with acoustic neuroma. *Neuroscience Research*.

[30] Uehara N., Tanimoto H., Nishikawa T. (2011). Vestibular dysfunction and compensation after removal of acoustic neuroma. *Journal of Vestibular Research: Equilibrium and Orientation*.

[31] Dumas G., Lion A., Gauchard G. C., Herpin G., Magnusson M., Perrin P. P. (2013). Clinical interest of postural and vestibulo-ocular reflex changes induced by cervical muscles and skull vibration in compensated unilateral vestibular lesion patients. *Journal of Vestibular Research: Equilibrium & Orientation*.

[32] Ceyte H., Cian C., Zory R., Barraud P.-A., Roux A., Guerraz M. (2007). Effect of Achilles tendon vibration on postural orientation. *Neuroscience Letters*.

[33] Maier J. X., Groh J. M. (2009). Multisensory guidance of orienting behavior. *Hearing Research*.

[34] Folk C. L., Ester E. F., Troemel K. (2009). How to keep attention from straying: Get engaged!. *Psychonomic Bulletin and Review*.

[35] Ho C., Santangelo V., Spence C. (2009). Multisensory warning signals: when spatial correspondence matters. *Experimental Brain Research*.

[36] Colonius H., Arndt P. (2001). A two-stage model for visual-auditory interaction in saccadic latencies. *Perception and Psychophysics*.

[37] Corneil B. D., Van Wanrooij M., Munoz D. P., Van Opstal A. J. (2002). Auditory-visual interactions subserving goal-directed saccades in a complex scene. *Journal of Neurophysiology*.

[38] Cohen S. M., Labadie R. F., Dietrich M. S., Haynes D. S. (2004). Quality of life in hearing-impaired adults: the role of cochlear implants and hearing aids. *Otolaryngology—Head and Neck Surgery*.

[39] Zhao F., Bai Z., Stephens D. (2008). The relationship between changes in self-rated quality of life after cochlear implantation and changes in individual complaints. *Clinical Otolaryngology*.

[40] Tjernström F., Bagher A., Fransson P.-A., Magnusson M. (2010). Short and long-term postural learning to withstand galvanic vestibular perturbations. *Journal of Vestibular Research: Equilibrium and Orientation*.

[41] Tjernström F., Fransson P.-A., Magnusson M. (2005). Improved postural control through repetition and consolidation. *Journal of Vestibular Research: Equilibrium and Orientation*.

[42] Wrisley D. M., Stephens M. J., Mosley S., Wojnowski A., Duffy J., Burkard R. (2007). Learning effects of repetitive administrations of the sensory organization test in healthy young adults. *Archives of Physical Medicine and Rehabilitation*.

